# Efficacy of RyR2 inhibitor EL20 in induced pluripotent stem cell‐derived cardiomyocytes from a patient with catecholaminergic polymorphic ventricular tachycardia

**DOI:** 10.1111/jcmm.16521

**Published:** 2021-06-10

**Authors:** Tarah A. Word, Ann P. Quick, Christina Y. Miyake, Mayra K. Shak, Xiaolu Pan, Jean J. Kim, Hugh D. Allen, Martha Sibrian‐Vazquez, Robert M. Strongin, Andrew P. Landstrom, Xander H. T. Wehrens

**Affiliations:** ^1^ Department of Molecular Physiology & Biophysics Cardiovascular Research Institute Baylor College of Medicine Houston TX USA; ^2^ Section of Cardiology Department of Pediatrics Baylor College of Medicine Houston TX USA; ^3^ Department of Molecular & Cellular Biology Stem Cells and Regenerative Medicine Center Baylor College of Medicine Houston TX USA; ^4^ Elex Biotech Inc Portland OR USA; ^5^ Department of Chemistry Portland State University Portland OR USA; ^6^ Department of Pediatrics Division of Cardiology Duke University School of Medicine Durham NC USA; ^7^ Department of Cell Biology Duke University School of Medicine Durham NC USA; ^8^ Department of Medicine Section of Cardiology Baylor College of Medicine Houston TX USA; ^9^ Department of Neuroscience Section of Cardiology Baylor College of Medicine Houston TX USA; ^10^ Center for Space Medicine Baylor College of Medicine Houston TX USA

**Keywords:** Catecholaminergic polymorphic ventricular tachycardia, induced pluripotent stem cells, ryanodine receptors, RyR2, tetracaine, ventricular arrhythmia

## Abstract

Catecholaminergic polymorphic ventricular tachycardia (CPVT) is an inherited cardiac arrhythmia syndrome that often leads to sudden cardiac death. The most common form of CPVT is caused by autosomal‐dominant variants in the cardiac ryanodine receptor type‐2 (*RYR2)* gene. Mutations in *RYR2* promote calcium (Ca^2+^) leak from the sarcoplasmic reticulum (SR), triggering lethal arrhythmias. Recently, it was demonstrated that tetracaine derivative EL20 specifically inhibits mutant RyR2, normalizes Ca^2+^ handling and suppresses arrhythmias in a CPVT mouse model. The objective of this study was to determine whether EL20 normalizes SR Ca^2+^ handling and arrhythmic events in induced pluripotent stem cell‐derived cardiomyocytes (iPSC‐CMs) from a CPVT patient. Blood samples from a child carrying RyR2 variant RyR2 variant Arg‐176‐Glu (R176Q) and a mutation‐negative relative were reprogrammed into iPSCs using a Sendai virus system. iPSC‐CMs were derived using the Stemdiff^TM^ kit. Confocal Ca^2+^ imaging was used to quantify RyR2 activity in the absence and presence of EL20. iPSC‐CMs harbouring the R176Q variant demonstrated spontaneous SR Ca^2+^ release events, whereas administration of EL20 diminished these abnormal events at low nanomolar concentrations (IC_50_ = 82 nM). Importantly, treatment with EL20 did not have any adverse effects on systolic Ca^2+^ handling in control iPSC‐CMs. Our results show for the first time that tetracaine derivative EL20 normalized SR Ca^2+^ handling and suppresses arrhythmogenic activity in iPSC‐CMs derived from a CPVT patient. Hence, this study confirms that this RyR2‐inhibitor represents a promising therapeutic candidate for treatment of CPVT.

## 1 INTRODUCTION

Catecholaminergic polymorphic ventricular tachycardia (CPVT) is a life‐threatening inherited cardiac arrhythmia syndrome that can lead to sudden cardiac arrest and death at a very high rate if left untreated. It is characterized by polymorphic and/or bi‐directional ventricular tachycardia (VT) that is triggered by β‐adrenergic stimulation associated with vigorous exercise or emotional stress.^1^ The prevalence of CVPT is estimated to be around 1:5000 to 1:10 000; both sexes are affected equally. The most common form of CPVT is caused by autosomal‐dominant variants in the *RYR2* gene encoding the cardiac ryanodine receptor type‐2.^2^, ^3^ The RyR2 channel releases Ca^2+^ from the sarcoplasmic reticulum (SR) in response to electrical excitation of the plasma membrane, which leads to influx of Ca^2+^ via the L‐type Ca^2+^ channel. Variants in *RYR2* promote diastolic SR Ca^2+^ leak which causes triggered activity and can initiate arrhythmias seen in CPVT.^1^, ^4^

Although CVPT is one of the most lethal forms of inherited cardiac disease, current treatment options remain limited. β‐adrenoreceptor blockers only control symptoms and suppress arrhythmias in about 30%‐50% of patients.^5^ Flecainide—a class 1C Na^+^ channel blocker that also normalizes RyR2 activity—has shown potential in CPVT patients but also has a black box warning for patients with any kind of structural heart disease.^6^, ^7^ Implantable cardioverter‐defibrillators (ICDs) are used in the event of severe or recurrent arrhythmias, but ICD discharges may further aggravate catecholamine release and lead to electrical storm.^8^ Hence, there remains an unmet need to develop new pharmacological or biological agents for the treatment of CPVT that specifically correct disease‐causing molecular defects.

Several classes of drugs have been developed that target mutant or dysfunctional RyR2 channels.^9^ These include, among others, benzothiazepine derivatives (K201, S107),^10^, ^11^ tetracaine derivatives (EL9, EL20),^12^, ^13^ class 1C anti‐arrhythmic drug derivatives of flecainide and propafenone,^14^ hydantoin derivatives (such as dantrolene),^15^ non‐beta blocking carvedilol derivatives^16^ and unnatural verticilide enantiomers.^17^ The efficacy of these compounds have been evaluated in various knock‐in mouse models carrying RyR2 variants or calsequestrin‐2 variants/knockout.^11^, ^12^, ^14^, ^18^ For example, mice heterozygous for RyR2 variant RyR2 variant Arg‐176‐Glu (R176Q) are susceptible to ventricular tachycardia after injection of caffeine and epinephrine, or following programmed ventricular stimulation and administration of β‐adrenergic receptor agonist isoproterenol (ISO).^18^

We recently demonstrated that tetracaine derivative EL20 can normalize aberrant SR Ca^2+^ leak associated with CPVT in the R176Q mouse model.^13^ The half‐maximum inhibitory concentration of EL20 in mouse myocytes was 35.4 mmol/L,^13^ which was an order of magnitude better than that of another well‐studied RyR2 inhibitor JTV519 (K201).^12^ This tetracaine derivative does not block Na^+^ or I_Kr_ channels within the effective concentration range used to inhibit RyR2 channels and has a favourable plasma stability profile (data not shown). In addition, tetracaine derivatives appear to work for all gain‐of‐function RyR2 variants, which represent the vast majority (>95%) of all known CPVT‐causing variants (>150 described to date). It remains unclear, however, whether a compound like EL20 also normalizes CPVT‐mutant RyR2 in human cardiac myocytes. Therefore, we developed induced pluripotent stem cell‐derived cardiomyocytes (iPSC‐CMs) from a CPVT patient carrying the same R176Q variant in *RYR2*. Confocal imaging revealed that EL20 effectively suppresses the enhanced Ca^2+^ spark frequency observed in iPSC‐CMs from this CPVT patient. These findings suggest that this RyR2‐inhibitor represents a promising therapeutic candidate for treatment of CPVT patients.

## 2 MATERIALS AND METHODS

### 2.1 Human subject recruitment

All studies were approved by the Baylor College of Medicine IRB and follow the Declaration of Helsinki guidelines. Subjects were evaluated by a paediatric electrophysiologist and a history, physical examination, echocardiogram, electrocardiogram and ancillary testing were performed. Following written receipt of informed consent, whole blood was obtained from the proband and kindred.

### 2.2 Cardiac differentiation of iPSCs

iPSCs were differentiated into iPSC‐CMs using the STEMdiff^TM^ CM differentiation kit (Stemcell Technologies) according to the company protocol (Document #DX21496). See Supplemental Methods for details.

### 2.3 Dissociation and plating of mature iPSC‐CMs

Spontaneously beating CMs were dissociated using the STEMDiff^TM^ Cardiomyocyte Dissociation Kit (Stemcell Technologies; Document #DX21497). See Supplemental Methods for details.

### 2.4 EL20 synthesis

The tetracaine derivative, 2‐(diethylamino)ethyl 4‐butlamino)‐2methoxybenzoate, also known as EL20, was synthesized using a two‐step synthesis route as previously described by Klipp et al.^13^

### 2.5 Ca^2+^ imaging of iPSC‐CMs and drug treatment using EL20

For drug assay studies, iPSC‐CMs were incubated with drug (concentration range 0.5 nmol/L‐5 μmol/L) for 30 minutes at 37°C. Ca^2+^ sparks were recorded in line‐scan mode using a LSM880 confocal microscope (Carl Zeiss). CaSpF was assessed with ImageJ software using SparkMaster plugin. See Supplemental Methods for details.

### 2.6 Statistical analysis

Results are expressed as mean ± SEM. For clustered data in which continuous variables were observed, the generalized estimating equation was used via SPSS version 24 (IBM) or Prism 8 (GraphPad) using an unpaired Student's *t* test or ANOVA, after performing the D’Agostino‐Pearson normality test for normal data distribution. For multiple group comparison, two‐way ANOVA followed by Tukey's post‐test was used. *P* < .05 was considered statistically significant.

## 3 RESULTS

### 3.1 Clinical evaluation of CPVT proband

**FIGURE 1:**
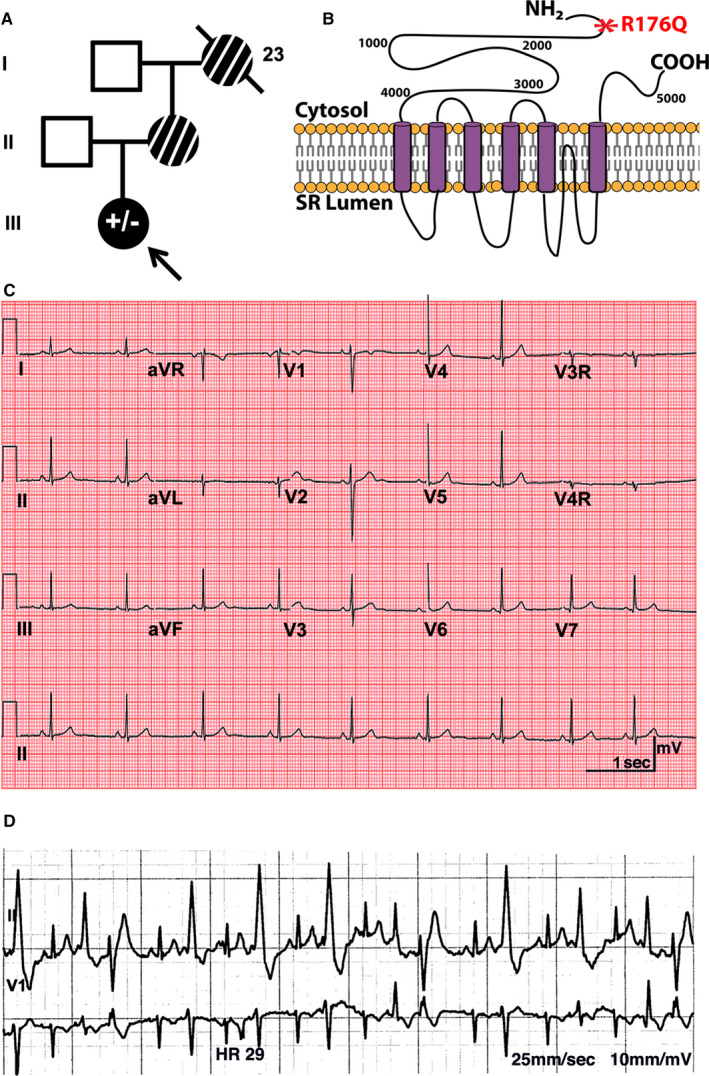
Pedigree and clinical phenotype of catecholaminergic polymorphic ventricular tachycardia (CPVT) patient: A, 14‐year‐old proband and kindred. The CPVT proband is identified by a black arrow, and the R176Q variant in RYR2 is denoted by a filled black symbol (± represents heterozygosity for the R176Q variant). Unaffected family members are shown as open symbols. Family members with a history of sudden cardiac arrest who were not genetically tested are shown as striped symbols. Line through symbol represents deceased family member. B, Schematic of RyR2 protein highlighting R176Q mutation (red). C, Standard resting 12‐lead ECG of the proband showing normal activity. D, Episode of polymorphic ventricular ectopy following epinephrine challenge in proband

The patient was previously a seemingly healthy female with a family history of sudden cardiac death (Figure 1A,B). Her maternal grandmother died postpartum at the age of 23 years, after giving birth to the proband's mother. The proband's mother suffered an aborted cardiac arrest at the age of 19 and again at the age of 35 that consequently left her in a persistent vegetative state. The proband underwent cardiac evaluation at the age of 14 and was found to have a structurally normal heart with normal biventricular function by echocardiogram (Figure S1). Her 12‐lead resting ECGs revealed a normal heart rate (54 beats per minute), normal PR interval (144 ms), normal QT interval (410 ms) and a normal corrected QT interval (QTc of 389 ms) (Figure 1C). She had no prior history of syncope and denied any cardiac symptoms. A few months after initial evaluation, the patient suffered an aborted cardiac arrest after running home from school. She was found to be in ventricular fibrillation with return of spontaneous circulation after successful defibrillation. During hospital admission, she was noted to have bi‐directional premature ventricular contractions during periods of stimulation. Due to suspicion of CPVT, she underwent an epinephrine challenge which revealed bi‐directional VT and was subsequently diagnosed with CPVT (Figure 1D). Genetic testing confirmed a heterozygous pathogenic missense mutation in *RYR2* (c.527G>A, p.Arg176Gln and R176Q). The CPVT proband is currently being treated with a β‐blocker and has an ICD. Her father (41‐year‐old) is an unaffected family member and is considered a healthy, mutation‐negative control.

### 3.2 Development, characterization and cardiac differentiation of iPSCs

Blood from the CPVT proband and mutation‐negative father was collected, and peripheral blood mononuclear cells (PBMCs) were extracted and subsequently reprogrammed into iPSCs using Sendai viruses (Figure S2A‐D). Both cell lines were karyotyped and exhibited normal chromosomal architectures (Figure S2E). The cells were genetically sequenced and the mutant variant (G > A) was found to be present in the proband‐derived iPSCs but not in those derived from the control individual (Figure S2F). The pluripotency of the cells was validated using fluorescence‐activated cell sorting (FACS) (Figure S3). CPVT proband and control‐derived iPSCs were differentiated into CMs that contracted spontaneously as coordinated sheets observed under light microscopy (Figure S4A,B). iPSC‐CMs dissociated into single cells from both lines maintained spontaneous contraction. qPCR demonstrated down‐regulation of the pluripotency markers and SOX2 (Tables S1 and S2; Figure S4C) and up‐regulation of the cardiac and Ca^2+^ handling markers (Table S2; Figure S4C). Immunofluorescence exhibited positive staining for similar cardiac and ca^2+^ handling markers (Table S3; Figure S4D).

### 3.3 CPVT patient‐derived iPSC‐CMs exhibit spontaneous and evoked Ca^2+^ transients

**FIGURE 2:**
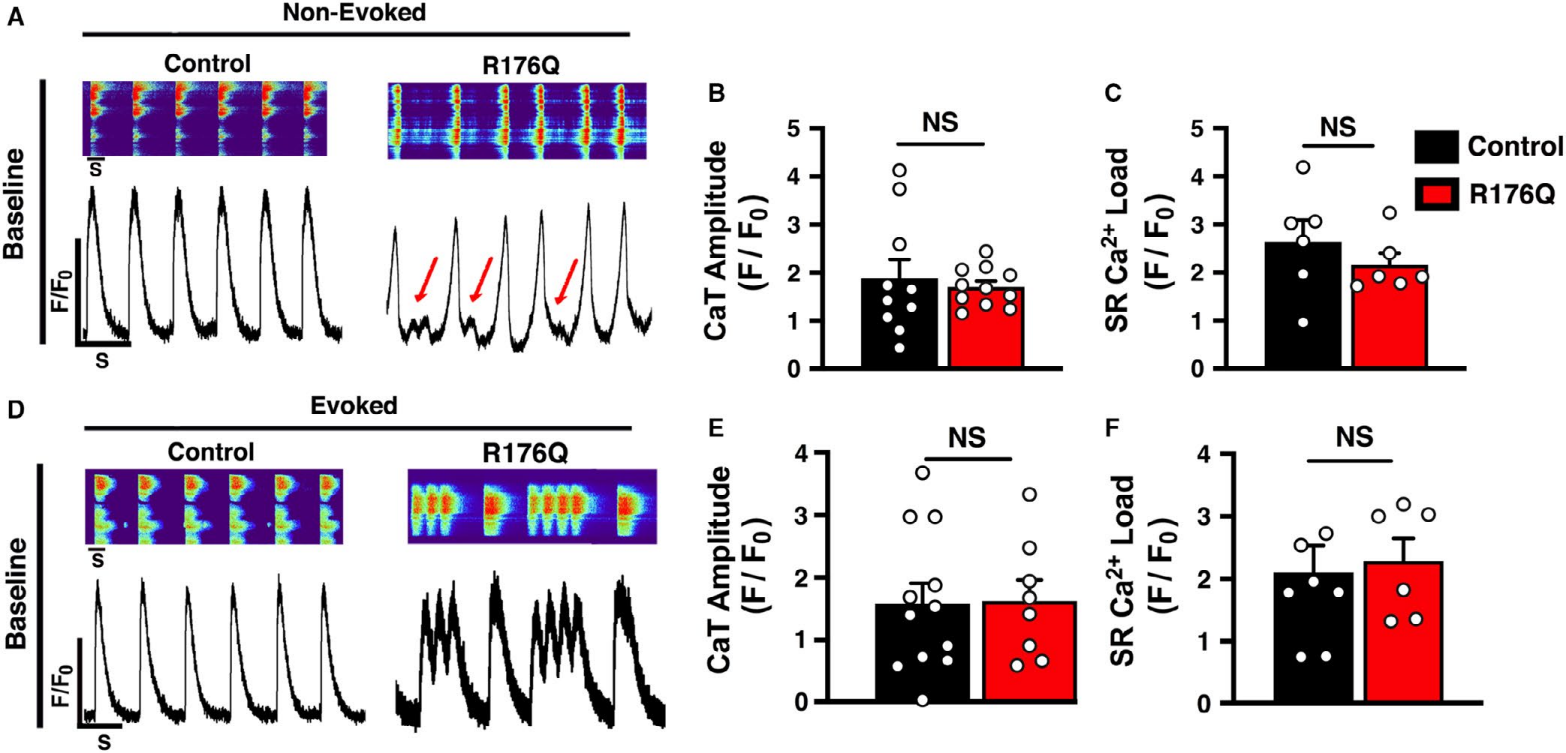
Systolic Ca^2+^ transients in control and R176Q proband‐derived induced pluripotent stem cell‐derived cardiomyocytes (iPSC‐CMs) under baseline conditions. A, Confocal line‐scan images (top) and Ca^2+^ transients (bottom) in iPSC‐CMs from the proband with the R176Q variant and healthy control under baseline conditions. Red arrows indicate spontaneous SR Ca^2+^ release events that developed during the diastolic phase in PSC‐CMs from the proband. Bar graph summarizing non‐evoked B, Ca^2+^ transient amplitude and C, 10 mmol/L caffeine‐induced SR Load under baseline conditions of control (black) and R176Q (red) iPSC‐CMs. D, Confocal line‐scan images (upper panel) and transients (bottom panel) showing intracellular Ca^2+^ handling loaded with fluro‐4‐AM of evoked (1‐Hz field stimulation) control vs R176Q iPSC‐CMs at baseline. DADs were further exacerbated in stimulated R176Q iPSC‐CMs. Bar graph summarizing evoked E, Ca^2+^ transient amplitude and F, 10 mmol/L caffeine‐induced SR Load under baseline conditions. Open circles represent individual cells. Data presented as mean ± SEM (NS = not statistically significant)

Previous studies revealed that beating clusters of iPSC‐CMs contain a mixture of ventricular, atrial and sinus node‐like cell populations based on their action potential (AP) phenotypes.^19^ Here, we used confocal Ca^2+^ imaging to characterized intracellular Ca^2+^ handling in dissociated iPSC‐CMs that exhibited a ventricular‐type morphology. About 50% of iPSC‐CMs exhibited non‐evoked, spontaneous rhythmic beating without electrical stimulation (Figure 2A). The average spontaneous beating rate of non‐evoked iPSC‐CMs with the R176Q mutation was 80.8 ± 8.4/min vs control iPSC‐CMs 41.6 ± 8.4/min (*P* = .002) consistent with being arrhythmic (Figure S5). In addition, 60% (6 out of 10 cells) of non‐evoked R176Q iPSC‐CM exhibited irregular Ca^2+^ waveforms following completion of the Ca^2+^ transient, thus resembling events that could initiate delayed afterdepolarizations (DADs). The amplitudes of non‐evoked Ca^2+^ transients were similar between the groups (F/F_0_ in R176Q: 1.7 ± 0.35 vs control: 1.9 ± 0.33; *P* = .64) (Figure 2B). The SR Ca^2+^ load assessed by a caffeine dump protocol was found to be similar between both groups (F/F_0_ in R176Q: 2.2 ± 0.24 vs control: 2.6 ± 0.88 and *P* = .37) (Figure 2C). Next, we performed electrical field stimulation to characterize iPSC‐CMs that were not spontaneously active under baseline conditions. About 75% of the iPSC‐CMs that did not spontaneously beat at baseline followed the 1‐Hz pacing train (Figure 2D); the remainder of the cells did not exhibit SR Ca^2+^ release upon electrical stimulation. While similar number of iPSC‐CMs with the R176Q variant responded to electrical stimulation, 63% of those iPSC‐CMs exhibited aberrant SR Ca^2+^ release patterns characterized by Ca^2+^ oscillations during the Ca^2+^ transient decay phase, that resemble those seen in conjunction with early afterdepolarizations (EADs). Finally, the amplitude of the Ca^2+^ transients (F/F_0_ in R176Q: 1.6 ± 0.21 vs control: 1.6 ± 0.44; *P* = .94) (Figure 2E) and total SR Ca^2+^ load (F/F_0_ in R176Q: 2.3 ± 0.36 vs control: 2.1 ± 0.43; *P* = .76) (Figure 2F) was similar amongst the groups. Together, these results show that systolic SR Ca^2+^ handling was similar between R176Q and control iPSC‐CMS, while aberrant diastolic SR Ca^2+^ release events were seen exclusively in iPSC‐CM from the CPVT patient.

### 3.4 Isoproterenol generates EADs in R176Q iPSC‐CMs spontaneous and evoked Ca^2+^ transients

**FIGURE 3:**
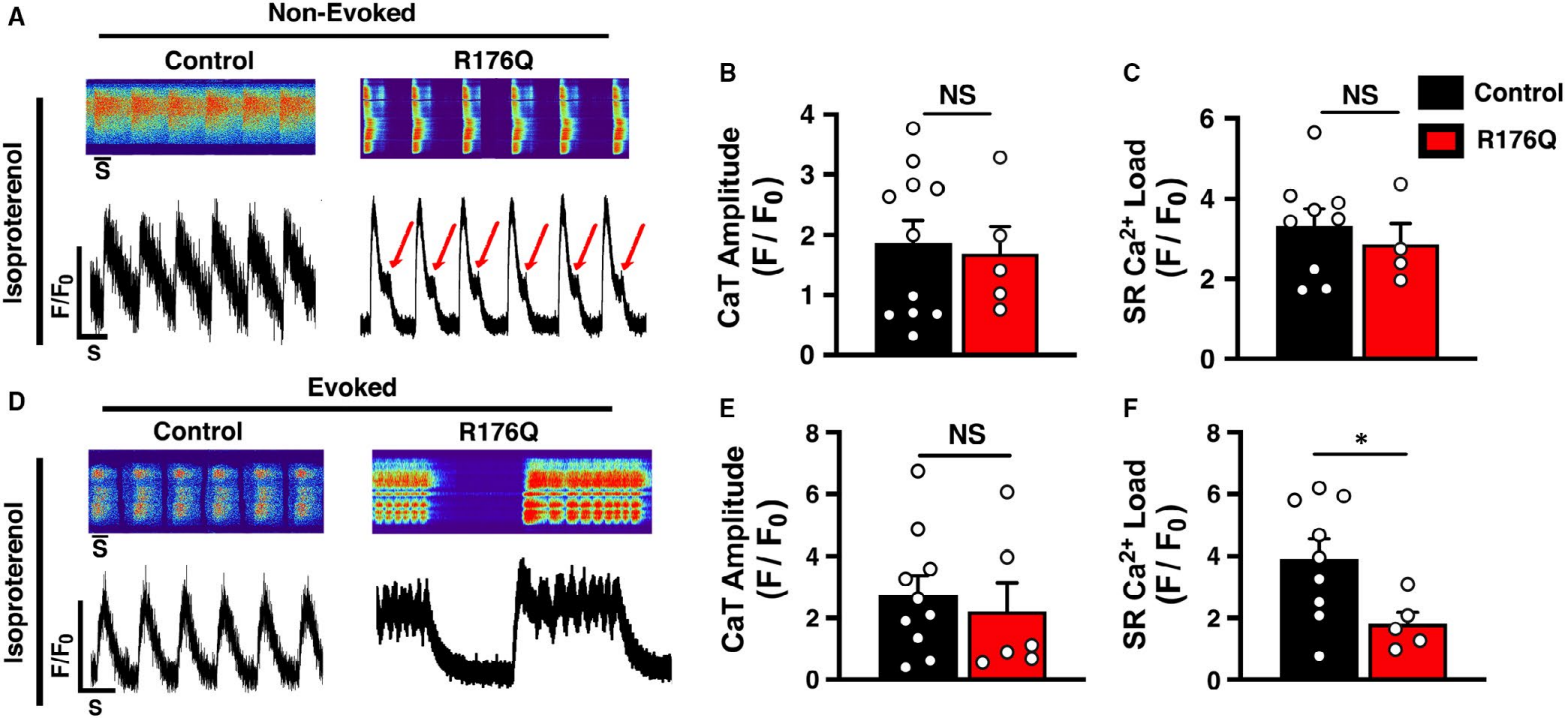
Effects of isoproterenol on systolic Ca^2+^ transients in control and R176Q‐proband‐derived induced pluripotent stem cell‐derived cardiomyocytes (iPSC‐CMs) under baseline conditions. A, Confocal line‐scan images (top) and Ca^2+^ transients (bottom) in iPSC‐CMs from the proband with the R176Q variant and healthy control in the presence of 100‐nmol/L isoproterenol (ISO). Red arrows indicate spontaneous SR Ca^2+^ release events that developed during the diastolic phase in PSC‐CMs from the proband. Bar graph summarizing non‐evoked B, Ca^2+^ transient amplitude and C, 10 mmol/L caffeine‐induced SR Load under baseline conditions of control (black) and R176Q (red) iPSC‐CMs. D, Confocal line‐scan images (upper panel) and transients (bottom panel) showing intracellular Ca^2+^ handling loaded with fluro‐4‐AM of evoked (1‐Hz field stimulation) control vs R176Q iPSC‐CMs in the presence of 100‐nmol/L isoproterenol (ISO). SR Ca^2+^ oscillations were further exacerbated in stimulated R176Q iPSC‐CMs. Bar graph summarizing evoked E, Ca^2+^ transient amplitude and F, 10 mmol/L caffeine‐induced SR Ca^2+^ load in the presence of 100‐nmol/L ISO. Open circles represent individual cells. Data presented as mean ± SEM (*P < .05; NS = not statistically significant)

To simulate β‐adrenergic stimulation conditions that provoke arrhythmias in CPVT patients, 100 nmol/L isoproterenol (ISO) was added to the iPSC‐CMs. Addition of ISO led to a positive chronotropic response compared to baseline conditions, as typically seen in healthy iPSC‐CMs.^20^ The average spontaneous beating rate of non‐evoked iPSC‐CMs with the R176Q mutation was 122.3 ± 18.1 /minutes vs control iPSC‐CMs 35.4 ± 5.2 (*P* = .002). 53% of the iPSC‐CMs with the R176Q variant exhibited abnormal SR Ca^2+^ release events during the final phase of SR Ca^2+^ reuptake, compared to 0% of the control cells (*P* < .05) (Figure 3A). The amplitudes of non‐evoked Ca^2+^ transients were similar between the groups (F/F_0_ in R176Q: 1.7 ± 0.45 vs control: 1.9 ± 0.37; *P* = .78) (Figure 3B). The SR Ca^2+^ load was found to be similar between both groups (F/F_0_ in R176Q: 2.7 ± 0.52 vs control: 3.3 ± 0.42 and *P* = .53) (Figure 3C).

About 75% of the iPSC‐CMs that did not spontaneously beat did generate SR Ca^2+^ transients following the 1‐Hz pacing train (Figure 3D). While similar number of iPSC‐CMs with the R176Q variant responded to electrical stimulation, 71% (5 of 7) of those iPSC‐CMs exhibited distinct oscillating SR Ca^2+^ release patterns that originated early during the Ca^2+^ transient recovery phase. These abnormalities were not observed in any of the control iPSC‐CMs after ISO stimulation. The amplitude of the Ca^2+^ transients (F/F_0_ in R176Q: 2.2 ± 1.5 vs control: 2.7 ± 0.89; *P* = .63) was similar amongst the two groups (Figure 3E). However, the SR Ca^2+^ load was significantly lower in R176Q iPSC‐CM (F/F_0_: 1.8 ± 0.37) compared with control iPSC‐CMs (F/F_0_: 3.9 ± 0.64; *P* = .039), suggesting that the oscillating SR Ca^2+^ release events interfere with SR Ca^2+^ refilling.

### 3.5 Effect of EL20 Ca^2+^ sparks in iPSC‐CMs

**FIGURE 4:**
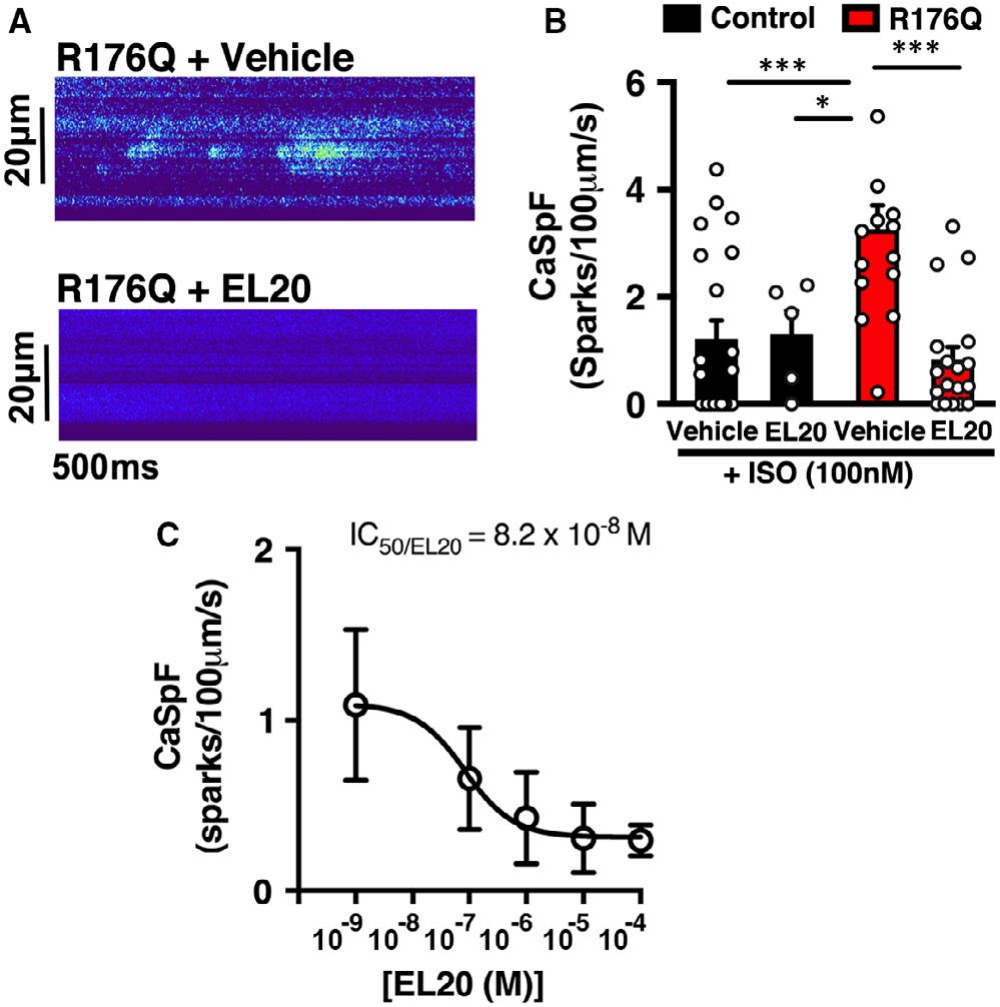
EL20 suppresses abnormal SR Ca^2+^ release events in induced pluripotent stem cell‐derived cardiomyocytes (iPSC‐CMs) derived from the catecholaminergic polymorphic ventricular tachycardia patient (CPVT) proband. A, Representative recordings of Ca^2+^ sparks in iPSC‐CMs from the CPVT proband in the absence and presence of EL20 (500‐nmol/L). B, Summary data showing that EL20 reduced the Ca^2+^ spark frequency (CaSpF) in stimulated iPSC‐CM compared to vehicle treatment. C, Dose‐dependence of Ca^2+^ spark frequency inhibition and IC_50_ determination of EL20 (n = 9‐10 cells per group). Open circles represent individual cells. Data presented as mean ± SEM (*P < .05, **P < .01, and ***P < .001)

Ca^2+^ sparks were recorded using line‐scan mode on a LSM880 confocal microscope to determine the diastolic activity of RyR2 in iPSC‐CMs following a 1‐Hz conditioning pacing train. Because spontaneous contractions in the non‐evoked iPSC‐CM population can potentially confound diastolic Ca^2+^ releases, Ca^2+^ imaging was done only under evoked pacing conditions to profile the drug responses of EL20 in vitro. In the presence of 100 nmol/L ISO, iPSC with the R176Q mutation exhibited a higher Ca^2+^ spark frequency (CaSpF) (3.6 ± 0.45 sparks/100 μm/s) compared with iPSC from the control (1.2 ± 0.34 sparks/100 μm/s; *P* < .001) (Figure 4A,B). Next, a screening dose of 500 nmol/L of EL20 was applied to the cells to determine the effects of this RyR2 inhibitor on diastolic SR Ca^2+^ release events. In control iPSC‐CMs, the CaSpF was not significantly affected by the addition of EL20 (1.3 ± 0.44 sparks/100 μm/s; *P* = .92 vs vehicle). In contrast, in R176Q iPSC‐CMs, EL20 significantly reduced the CaSpF by 78% (to 0.8 ± 0.45 sparks/100 μm/s; *P* < .001 vs vehicle).

### 3.6 Dose‐response of Ca^2+^ spark inhibition by EL20

To get a better sense of what drug concentrations are needed to inhibit mutant RyR2 channels, we assessed the effects of different doses of EL20 on CaSpF in evoked iPSC‐CMs derived from the CPVT proband. The dose‐response curve, which is based on inhibition of the CaSpF, was determined at concentrations ranging from 10^−9^ up to 10^−4 ^mol/L (Figure 4C). Results from the sigmoidal dose‐response curve determined that the half maximal inhibitory concentration (IC_50_) of EL20 is 8.2 × 10^−8 ^mol/L. Hence, EL20 was found to inhibit excessive SR Ca^2+^ release at low nanomolar concentrations that are clinically relevant.

### 3.7 Absence of detrimental effects of EL20 on systolic SR Ca^2+^ handling

**FIGURE 5:**
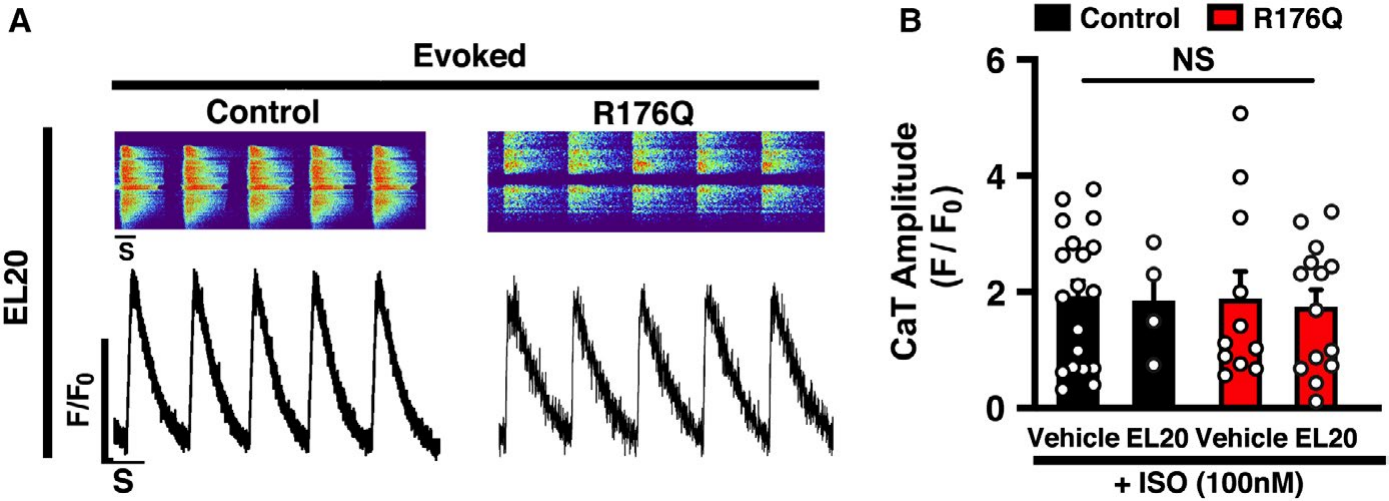
Absence of detrimental effects on evoked systolic SR Ca^2+^ transients by EL20. A, Confocal line‐scan images (top) and transients (bottom) showing intracellular Ca^2+^ transients recorded in induced pluripotent stem cell‐derived cardiomyocytes (iPSC‐CMs) loaded with fluro‐4‐AM during 1‐Hz field stimulation pacing in the presence 500‐nmol/L EL20. B, Bar graph summarizing the Ca^2+^ transient amplitude in iPSCs from both the catecholaminergic polymorphic ventricular tachycardia patient proband and control individual. Open circles represent individual cells. Data presented as mean ± SEM (NS = not statistically significant.)

Since EL20 inhibits aberrant SR Ca^2+^ release through mutant RyR2, it is possible that this drug has unwanted effects on systolic SR Ca^2+^ transients consequently leading to unwanted side effects.^21^ To address this concern, we measured the Ca^2+^ transient amplitude induced by field stimulation at 1‐Hz in iPSC‐CMs (Figure 5). The amplitude of the SR Ca^2+^ transient was not altered by EL20 in iPSC‐CMs from the control (F/F_0_: 1.8 ± 0.46) compared to control + vehicle (F/F_0_: 1.9 ± 0.27; *P* = .91). Importantly, EL20 also did not alter the Ca^2+^ transient amplitude in R176Q iPSC‐CMs (F/F_0_: 1.7 ± 0.20) compared to R176Q + vehicle (F/F_0_: 1.9 ± 0.46; *P* = .79). Interestingly, treatment with EL20 prevented to occurrence of pacing‐evoked Ca^2+^ oscillations (Figure 3D), consistent with inhibition of aberrant RyR2 SR Ca^2+^ leak. Thus, EL20 does not negatively affect the Ca^2+^ transient amplitude at the cellular level, as previously seen in the CPVT mouse model.^12^

## 4 DISCUSSION

Catecholaminergic polymorphic ventricular tachycardia remains a difficult to treat inherited cardiac disorder associated with arrhythmias provoked by adrenergic stress. As reviewed in great detail in this recent review article,^9^ various classes of anti‐arrhythmic drugs with RyR2 inhibitory properties have been developed and some show great promise for the treatment of CPVT. Patient‐derived induced pluripotent stem cell‐derived cardiomyocytes (hiPSC‐CMs) offer a powerful cellular model to evaluate the potential effectiveness and safety of experimental drugs in a preclinical human cell model.^22^ In the present study, we developed and characterized a novel iPSC‐CM cell line containing an N‐terminal variant R176Q in RyR2. Our studies revealed evidence for abnormal diastolic SR Ca^2+^ release events in spontaneously beating and paced single iPSC‐CMs containing the R176Q variant in RyR2. Moreover, tetracaine derivative EL20 was able to suppress these aberrant SR Ca^2+^ release events at low nanomolar doses, suggesting that EL20 and related compounds are promising agents for the treatment of CPVT patients.

### 4.1 Preclinical CPVT models based on iPSC‐CMs

Patient‐specific induced pluripotent stem cell‐derived cardiomyocytes have emerged as a powerful model of inherited arrhythmia disorders after the development of stem cell reprogramming by Yamanaka et al^23^ iPSC‐CMs containing various CPVT‐associated RyR2 variants have been generated and studies using confocal imaging and patch‐clamping approaches. Most studies to date revealed altered intracellular Ca^2+^ handling, in particular during final stages of repolarization, and increased Ca^2+^ spark frequencies, in iPSC‐CMs containing RyR2 variants.^24^ Consistent with clinical observations, the administration of β‐adrenergic agonists exacerbated these Ca^2+^ abnormalities in most studies. Jung et al^25^ investigated intracellular Ca^2+^ handling in great detail in CPVT iPSC‐CM and found evidence for increased diastolic SR Ca^2+^ leak as well as reduced SR Ca^2+^ store contents after tonic stimulation. Our studies revealed similar findings including an increased SR Ca^2+^ spark frequency and reduced SR Ca^2+^ load after β‐adrenergic stimulation. These data suggest that enhanced diastolic SR Ca^2+^ leak in particular during β‐adrenergic stimulation is the primary mechanism underlying arrhythmia formation, rather than SR Ca^2+^ overload which has been proposed as one mechanism of CPVT by some authors.^26^, ^27^

In this study, we developed iPSC‐CMs derived from a 14‐year‐old girl with the same N‐terminal RyR2 variant R176Q as the mouse model previously reported by us.^13^, ^18^ The R176Q variant caused a highly penetrant and severe phenotype in this affected family (Figure 1). The father, who is negative for the RyR2 variant, was the donor for the control iPSC‐CM cell line. Characterization of intracellular Ca^2+^ handling in the control iPSC‐CMs revealed no abnormalities, confirming the specificity and validity of the CPVT iPSC‐CM cell line. The iPSC‐CM containing the R176Q variant exhibited several distinct abnormalities in intracellular Ca^2+^ handling. In spontaneously beating as well as pacing cells, spontaneous SR Ca^2+^ release events were seen during the late and early Ca^2+^ reuptake phase of the Ca^2+^ transient. The events during early repolarization look similar to oscillatory events associated with EADs in other studies in which action potentials were measured in iPSC‐CMs.^28^, ^29^ These events more predominantly seen following b‐adrenergic stimulation. In addition, aberrant SR Ca^2+^ release events were observed after or during the final repolarization phase, consistent with DADs. These findings are consistent with prior studies and recapitulate the CPVT phenotype within the iPSC‐CM model.^28^ Patients with RyR2 variants can be susceptible to both EAD‐ and DAD‐mediated arrhythmia mechanisms because membrane voltage is strongly influenced by Ca^2+^‐sensitive ionic currents, and, conversely, cellular Ca^2+^ loading is strongly influenced by Ca^2+^ voltage‐dependent currents that can promote complex AP dynamics in the heart.^30^ The diastolic SR Ca^2+^ leak can activate the Na^+^/Ca^2+^‐exchanger, leading to a net inward Na^+^ current that can depolarize the plasma membrane leading to an arrhythmogenic beat.

### 4.2 EL20 prevents arrhythmogenic Ca^2+^ release events in CPVT iPSC‐CMs

Several prior studies showed that iPSC‐CMs derived from CPVT patients can be utilized to identify drug molecules that correct the cellular phenotypes associated with mutant RyR2.^24^ In prior work, we synthesized a novel tetracaine derivative known as EL20 and tested its pharmacological properties in the RyR2‐R176Q heterozygous mouse model of CPVT.^13^ EL20 prevented ventricular tachycardia in R176Q/+ mice. Another tetracaine derivative known as EL9 also suppressed VT in the R176Q/+ mouse model without adverse effects on cardiac contractility.^12^ In ventricular myocytes isolated from R176Q/+ mice, EL9 normalized the increased Ca^2+^ spark frequency with an IC_50_ of 13 nmol/L.^12^ These findings in ventricular myocytes isolated from a CPVT mouse model were quite similar to those reported for EL20 in iPSC‐CMs carrying the identical RyR2 variant R176Q. The present study represents the first to characterize the effects of a tetracaine derivative with RyR2 inhibiting activity (ie EL20) on intracellular Ca^2+^ handling properties in human cardiomyocytes. Our work reveals that EL20 can normalize intracellular Ca^2+^ handling at nanomolar concentrations and prevented cellular arrhythmogenesis in iPSC‐CM from a CPVT patient (Figure S6). On the other hand, EL20 did not exhibit unwanted effects in control iPSC‐CMs derived from the proband's father who is negative for the RyR2 variant. Moreover, EL20 did not affect systolic SR Ca^2+^ release as evidenced by unaltered Ca^2+^ transient amplitudes (Figure 5). Since there was no difference in SR Ca^2+^ load between control and R176Q cells under basal conditions (Figure 2C), we are quite confident that EL20 does not alter the SR Ca^2+^ load. Together, these findings indicate that EL20 directly inhibits diastolic SR Ca^2+^ release events through the R176Q mutant RyR2 channels.

Previous studies revealed that EL20 stabilizes hyperactive RyR2 channels.^13^ The molecular mechanism underlying the therapeutic effects of EL20 is believed to involve stabilization of the mutant RyR2 channel from which calmodulin has been dissociated as a result of the CPVT variant.^13^ On the other hand, EL20 did not alter the activity of wild‐type RyR2 channel to which normal amounts of calmodulin are bound.^13^ Future studies can be conducted to validate this model by overexpressing calmodulin or generating calmodulin variants that enhance its binding to RyR2 containing CPVT‐associated variants. Regardless, the observation that EL20 and likely other tetracaine derivatives only modulate RyR2 channels containing disease‐associated variants is promising and suggests that these drugs will lack significant side effects, in particular suppression of excitation‐contraction coupling and reduced cardiac contractility.

## 5 CONCLUSIONS

We found that iPSC‐CMs derived from a CPVT patient carrying RyR2 variant R176Q display irregular spontaneous Ca^2+^ release events that are well‐established triggers of arrhythmias. Our data show that EL20 eliminated cellular arrhythmias at nanomolar concentrations in the absence of obvious side effects on intracellular Ca^2+^ handling parameters examined in the iPSC‐CMs. Therefore, EL20 is a promising lead compound for further drug development efforts that could potentially provide novel treatment options for the orphan disease CPVT. RyR2 inhibitors such as EL20 directly target the disease‐causing molecular defect in patients with CPVT (ie leaky RyR2 channels) and could be more effective compared to beta blockers that work upstream in the beta‐adrenergic receptor signalling pathway that leads to RyR2 phosphorylation and exacerbation of SR Ca^2+^ leak during stress or exercise.^31^ In a patient with a very high arrhythmic burden, however, it might be beneficial to treat with both EL20 and beta blockers to prevent SR Ca^2+^ leak at multiple levels in the signalling pathway. In conclusion, our patient‐derived iPSC‐CM model offers a promising platform for further research into the pathophysiological mechanisms of CPVT, as well as a safe tool for screening and optimizing drug therapy using novel RyR2 inhibitors.

## CONFLICT OF INTEREST

RMS and XHTW are founding partners of Elex Biotech, a start‐up company that developed drug molecules to target ryanodine receptors for treatment of cardiac arrhythmias. Other authors have no conflicts related to this study.

## AUTHOR CONTRIBUTIONS

**Tarah Word:** Data curation (lead); Formal analysis (lead); Methodology (equal); Writing–original draft (lead). **Ann Quick:** Investigation (supporting); Writing–review and editing (supporting). **Christina Miyake:** Formal analysis (supporting); Investigation (supporting). **Mayra Shak:** Methodology (supporting). **Xialo Pan:** Methodology (supporting); Resources (supporting). **Jean Kim:** Methodology (supporting); Supervision (supporting). **Hugh Allen:** Supervision (supporting). **Martha Sibrian‐Vazquez:** Resources (supporting). **Robert Strongin:** Supervision (supporting); Validation (supporting); Writing–review and editing (supporting). **Andrew Landstrom:** Resources (supporting); Supervision (supporting); Writing–review and editing (supporting). **Xander H. Wehrens:** Conceptualization (lead); Project administration (lead); Resources (lead); Supervision (lead); Writing‐review & editing (lead).

## Supporting information

Supplementary Material

## References

[1] Wehrens XH, Lehnart SE, Huang F, et al. FKBP12.6 deficiency and defective calcium release channel (ryanodine receptor) function linked to exercise‐induced sudden cardiac death. Cell. 2003;113:829‐840. 12837242 10.1016/s0092-8674(03)00434-3

[2] Laitinen PJ, Brown KM, Piippo K, et al. Mutations of the cardiac ryanodine receptor (RyR2) gene in familial polymorphic ventricular tachycardia. Circulation. 2001;103:485‐490. 11157710 10.1161/01.cir.103.4.485

[3] Lehnart SE, Wehrens XH, Laitinen PJ, et al. Sudden death in familial polymorphic ventricular tachycardia associated with calcium release channel (ryanodine receptor) leak. Circulation. 2004;109:3208‐3214. 15197150 10.1161/01.CIR.0000132472.98675.EC

[4] Lehnart SE, Wehrens XH, Kushnir A, Marks AR. Cardiac ryanodine receptor function and regulation in heart disease. Ann N Y Acad Scin. 2004;1015:144‐159. 10.1196/annals.1302.012 15201156

[5] Imberti JF, Underwood K, Mazzanti A, Priori SG. Clinical challenges in catecholaminergic polymorphic ventricular tachycardia. Heart Lung Circ. 2016;25:777‐783. 26948768 10.1016/j.hlc.2016.01.012

[6] Watanabe H, Chopra N, Laver D, et al. Flecainide prevents catecholaminergic polymorphic ventricular tachycardia in mice and humans. Nat Med. 2009;15:380‐383. 19330009 10.1038/nm.1942 PMC2904954

[7] van der Werf C, Kannankeril PJ, Sacher F, et al. Flecainide therapy reduces exercise‐induced ventricular arrhythmias in patients with catecholaminergic polymorphic ventricular tachycardia. J Am Coll Cardiol. 2011;57:2244‐2254. 21616285 10.1016/j.jacc.2011.01.026 PMC3495585

[8] Roston TM, Jones K, Hawkins NM, et al. Implantable cardioverter‐defibrillator use in catecholaminergic polymorphic ventricular tachycardia: a systematic review. Heart Rhythm. 2018;15:1791‐1799. 30063211 10.1016/j.hrthm.2018.06.046

[9] Connell P, Word TA, Wehrens XHT. Targeting pathological leak of ryanodine receptors: preclinical progress and the potential impact on treatments for cardiac arrhythmias and heart failure. Expert Opin Ther Targets. 2020;24:25‐36. 31869254 10.1080/14728222.2020.1708326 PMC6956596

[10] Wehrens XH, Lehnart SE, Reiken SR, et al. Protection from cardiac arrhythmia through ryanodine receptor‐stabilizing protein calstabin2. Science. 2004;304:292‐296. 15073377 10.1126/science.1094301

[11] Fauconnier J, Thireau J, Reiken S, et al. Leaky RyR2 trigger ventricular arrhythmias in Duchenne muscular dystrophy. Proc Natl Acad Sci USA. 2010;107:1559‐1564. 20080623 10.1073/pnas.0908540107 PMC2824377

[12] Li N, Wang Q, Sibrian‐Vazquez M, et al. Treatment of catecholaminergic polymorphic ventricular tachycardia in mice using novel RyR2‐modifying drugs. Int J Cardiol. 2017;227:668‐673. 27838126 10.1016/j.ijcard.2016.10.078 PMC5164850

[13] Klipp RC, Li N, Wang Q, et al. EL20, a potent antiarrhythmic compound, selectively inhibits calmodulin‐deficient ryanodine receptor type 2. Heart Rhythm. 2018;15:578‐586. 29248564 10.1016/j.hrthm.2017.12.017 PMC5879004

[14] Galimberti ES, Knollmann BC. Efficacy and potency of class I antiarrhythmic drugs for suppression of Ca2+ waves in permeabilized myocytes lacking calsequestrin. J Mol Cell Cardiol. 2011;51:760‐768. 21798265 10.1016/j.yjmcc.2011.07.002 PMC3184367

[15] Kobayashi S, Yano M, Uchinoumi H, et al. Dantrolene, a therapeutic agent for malignant hyperthermia, inhibits catecholaminergic polymorphic ventricular tachycardia in a RyR2(R2474S/+) knock‐in mouse model. Circ J. 2010;74:2579‐2584. 20944434 10.1253/circj.cj-10-0680

[16] Smith CD, Wang A, Vembaiyan K, et al. Novel carvedilol analogues that suppress store‐overload‐induced Ca2+ release. J Med Chem. 2013;56:8626‐8655. 24124794 10.1021/jm401090a PMC3896386

[17] Batiste SM, Blackwell DJ, Kim K, et al. Unnatural verticilide enantiomer inhibits type 2 ryanodine receptor‐mediated calcium leak and is antiarrhythmic. Proc Natl Acad Sci USA. 2019;116:4810‐4815. 30792355 10.1073/pnas.1816685116 PMC6421472

[18] Kannankeril PJ, Mitchell BM, Goonasekera SA, et al. Mice with the R176Q cardiac ryanodine receptor mutation exhibit catecholamine‐induced ventricular tachycardia and cardiomyopathy. Proc Natl Acad Sci USA. 2006;103:12179‐12184. 16873551 10.1073/pnas.0600268103 PMC1567715

[19] Zhang XH, Morad M. Calcium signaling in human stem cell‐derived cardiomyocytes: evidence from normal subjects and CPVT afflicted patients. Cell Calcium. 2016;59:98‐107. 26725479 10.1016/j.ceca.2015.12.002 PMC4834249

[20] Schick R, Mekies LN, Shemer Y, et al. Functional abnormalities in induced pluripotent stem cell‐derived cardiomyocytes generated from titin‐mutated patients with dilated cardiomyopathy. PLoS One. 2018;13:e0205719. 30332462 10.1371/journal.pone.0205719 PMC6192629

[21] Ahola A, Polonen RP, Aalto‐Setala K, Hyttinen J. Simultaneous measurement of contraction and calcium transients in stem cell derived cardiomyocytes. Ann Biomed Eng. 2018;46:148‐158. 28975460 10.1007/s10439-017-1933-2 PMC5754453

[22] Juhola M, Penttinen K, Joutsijoki H, Aalto‐Setala K. Analysis of drug effects on iPSC cardiomyocytes with machine learning. Ann Biomed Eng. 2020;49(1):129‐138. 32367466 10.1007/s10439-020-02521-0 PMC7773623

[23] Takahashi K, Tanabe K, Ohnuki M, et al. Induction of pluripotent stem cells from adult human fibroblasts by defined factors. Cell. 2007;131:861‐872. 18035408 10.1016/j.cell.2007.11.019

[24] Bezzerides VJ, Zhang D, Pu WT. Modeling inherited arrhythmia disorders using induced pluripotent stem cell‐derived cardiomyocytes. Circ J. 2016;81:12‐21. 27916777 10.1253/circj.CJ-16-1113 PMC5436929

[25] Jung CB, Moretti A, Mederos y Schnitzler M, et al. Dantrolene rescues arrhythmogenic RYR2 defect in a patient‐specific stem cell model of catecholaminergic polymorphic ventricular tachycardia. EMBO Mol Med. 2012;4:180‐191. 22174035 10.1002/emmm.201100194 PMC3376852

[26] MacLennan DH, Chen SR. Store overload‐induced Ca2+ release as a triggering mechanism for CPVT and MH episodes caused by mutations in RYR and CASQ genes. J Physiol. 2009;587:3113‐3115. 19567749 10.1113/jphysiol.2009.172155 PMC2727021

[27] Dobrev D, Wehrens XH. Role of RyR2 phosphorylation in heart failure and arrhythmias: controversies around ryanodine receptor phosphorylation in cardiac disease. Circ Res. 2014;114:1311‐1319. 24723656 10.1161/CIRCRESAHA.114.300568 PMC4008932

[28] Kujala K, Paavola J, Lahti A, et al. Cell model of catecholaminergic polymorphic ventricular tachycardia reveals early and delayed afterdepolarizations. PLoS One. 2012;7:e44660. 22962621 10.1371/journal.pone.0044660 PMC3433449

[29] Zhao YT, Valdivia CR, Gurrola GB, et al. Arrhythmogenesis in a catecholaminergic polymorphic ventricular tachycardia mutation that depresses ryanodine receptor function. Proc Natl Acad Sci USA. 2015;112:E1669‐E1677. 25775566 10.1073/pnas.1419795112 PMC4386375

[30] Song Z, Ko CY, Nivala M, Weiss JN, Qu Z. Calcium‐voltage coupling in the genesis of early and delayed afterdepolarizations in cardiac myocytes. Biophys J. 2015;108:1908‐1921. 25902431 10.1016/j.bpj.2015.03.011 PMC4407256

[31] Chelu MG, Sarma S, Sood S, et al. Calmodulin kinase II‐mediated sarcoplasmic reticulum Ca2+ leak promotes atrial fibrillation in mice. J Clin Invest. 2009;119:1940‐1951. 19603549 10.1172/JCI37059 PMC2701862

