# Supplementary material for: Efficacy of RyR2 inhibitor EL20 in induced pluripotent stem cell‐derived cardiomyocytes from a patient with catecholaminergic polymorphic ventricular tachycardia
Source: J Cell Mol Med. 2021 Jun 10;25(13):6115–24. doi: 10.1111/jcmm.16521 (PMC8366453; doi:10.1111/jcmm.16521)
Supplement: Supplementary file 1 — Supplementary Material [file JCMM-25-6115-s001.docx]

**ONLINE SUPPLEMENTAL MATERIAL**

**Efficacy of RyR2 Inhibitor EL20 in Induced Pluripotent Stem Cell-Derived Cardiomyocytes from a Patient with CPVT**

Tarah A. Word, PhD, Ann P. Quick, PhD, Christina Miyake, MD, Mayra K. Shak, BS, Xiaolu Pan, MS, Jean J. Kim, PhD, Hugh D. Allen, MD, Martha Sibrian-Vazquez, Robert M. Strongin, Andrew P. Landstrom, MD, PhD, Xander H.T. Wehrens, MD, PhD

**SUPPLEMENTAL METHODS**

**Converting patient’s blood to PBMCs**

Whole blood samples from patients were collected and processed into peripheral mononuclear blood cell (PBMCs) lines in our laboratory using published protocols.^1, 2^ Approximately 8-10 mL of peripheral blood was processed within 24 hours of the blood draw. Isolation and expansion of PBMCs were done by diluting the peripheral blood into equal volume of 1x D-PBS without CaCl_2_ and MgCl_2_ (Thermo Fisher Scientific, Waltham, MA; #14200075) supplemented with 50 μL/mL RosetteSep^TM^ human monocyte enrichment cocktail (STEMCELL Technology, Vancouver, Canada; #15028), and then quickly poured into 15 mL of Lymphoprep (STEMCELL Technology, Vancouver, Canada; #07801) in a SepMate tube (STEMCELL Technology, Vancouver, Canada; #85450). The peripheral blood mixture was centrifuge for 10 min at 1,200 relative centrifugal field (RCF) to allow separation of the red blood cells from isolated PBMCs. The isolated PBMCs were poured into a new 50 mL conical tube, re-suspended in 15 mL of PBS, and centrifuge for an additional 10 minutes at 120 RCF. Supernatant was aspirated and purified PBMCs were re-suspend in 4 mL of Ethryroid Expansion Media (EEM) (STEMCELL Technology, Vancouver, Canada; #02694) supplemented with 100 μg/mL Primocin (Thermo Fischer Scientific, Waltham, MA; #Ant-pm-1), plated in a 12 well plate (~2.5 × 10^5^ PBMCs) and placed in incubator (37 ^o^C). EEM media was changed every two days for a total of 5 – 7 days.

**Reprogramming of PBMCs to iPSCs**

Successfully reprogramming of PBMCs into iPSCs lines was performed by the BCM Stem Cell Core and Applied Biological Materials (ABM),respectively. Peripheral blood mononuclear cells (PBMCs) were reprogrammed into iPSCs using the non-integrating CytoTune®-iPS 2.0 Reprogramming System (Thermo Fisher Scientific, Waltham, MA; #A16517). Sendai viruses were added at a multiplicity of infection (MOI) of 10:10:6 for the hKOS:c-Myc:Klf4 Sendai viruses. Three days after transduction, cells were cultured on hESC-qualified Matrigel® (Corning, Bedford, MA; #354277) in TeSR-E7 medium (STEMCELL Technologies, Vancouver, Canada; #05914) for 7 days, and then switched to TeSR-E8 medium (STEMCELL Technologies, Vancouver, Canada; #05990). After 21 days, individual iPSC colonies were mechanically isolated and expanded in a 37°C incubator with 5% CO2. After mechanically passaging for the first 3 passages, iPSCs were non-enzymatically passaged using ReLeSR (STEMCELL Technologies, Vancouver, Canada; #05872) at a split ratio of 1:4–1:6 and cryopreserved. Mycoplasma testing was performed with the MycoAlert PLUS kit (Lonza, Basel, Switzerland; #LT07-703) and the Lucetta^TM^ Luminometer (Lonza, Basel, Switzerland; #AAL-1002).

**Cell Culture of iPSCs**

iPSCs were cultured In 6-well plates that were coated with hESC-qualified Matrigel® prepared according to company protocol (1:200 dilution) using DMEM/F12 (STEMCELL Technologies, Vancouver, Canada; #36254) with daily media change using MTESR (STEMCELL Technologies, Vancouver, Canada; #05857) and 1% Penicillin-Streptomycin (Thermo Fisher Scientific, Waltham, MA; #15070063). Cryopreserved iPSCs thawed for culturing were supplemented with 10μM Rho kinase inhibitor Y-27632 (STEMCELL Technologies, Vancouver, Canada; #72302) for 24 hours. iPSC lines were passaged once cells reached ~70-75% confluency and by washing cells 1x with D-PBS without CaCl_2_ and MgCl_2_, followed by incubating the cells with ReLeSR^TM^ (STEMCELL Technologies, Vancouver, Canada; #05872) at room temperature for 1 minute. To allow full detachment of iPSCs, ReLeSR^TM^ was removed and cells were incubated at 37^o^C for ~4 minutes. iPSCs were re-suspended in MTESR and then split (1:3 dilution) on a 6-well plate coated with hESC-qualified Matrigel^®^ and maintained at 37^o^C, 5% CO_2_, and 90% humidity.

**Karyotyping**

Cytogenetic analysis was performed using standard protocols. Several clones from each patient were isolated, expanded, and characterized by G-banded karyotyping performed by Baylor Miraca Genetics Laboratories.

**Flow cytometry**

Flow cytometry of live mitotic cell cultures performed by the BCM Human Stem Cell Core and Applied Biological Materials (ABM). Pluripotency of iPSCs were characterized for SSEA3, SSEA4, OCT4, NANOG, TRA-1-61, and/or TRA-1-80. Cells were dissociated with Accutase (STEMCELL Technologies, Vancouver, Canada; #07920) for 10 minutes at 37°C, triturated, and passed through a 40 μm cell strainer. Cells were then washed twice with stain buffer (BD Biosciences, San Jose, CA; #554656) and resuspended at a maximum concentration of 5 × 10^6^ cells per 100 μL. Cells were fixed for 30 minutes at RT with BD Cytofix Fixation Buffer (BD Biosciences, San Jose, CA; #554655). The cells were then washed twice with stain buffer and permeabilized with BD Phosflow Perm Buffer III (BD Biosciences, San Jose, CA; #558050) for 30 minutes on ice. Cells were then washed twice with stain buffer. Antibodies were added at the dilution in 100 μL of cell suspension, as described.^3^ Cells were stained with primary antibodies for 1 hour on ice, washed, and resuspended in stain buffer. Cells were analyzed by an LSR II flow cytometer (BD Biosciences, San Jose, CA). All expanded clones were confirmed to be negative for mycoplasma.

**Validation of iPSCs by Sanger Sequencing**

Genomic DNA of iPSCs were extracted by DNeasy Blood and Tissue Kits (Qiagen, Hilden, Germany; #69504). Polymerase chain reaction was used to amplify the region of mutation on *RYR2* MyTaq Red Mix (Bioline, London, UK; #Bio-25043). Primers are listed in **Supplemental** **Table 1**. WT or R176Q mutation was validated by Sanger sequencing.

**Cardiac Differentiation of iPSCs**

iPSCs were differentiated into functional cardiomyocytes (iPSC-CMs) using STEMdiff^TM^ CM differentiation kit (Stemcell Technologies, Vancouver, Canada; #05010) according to the company protocol (document #DX21496). Briefly, iPSC lines were cultured in MTESR and 1% Penicillin-Streptomycin) media until the cells achieved a monolayer that reached ~95% confluency; referred to as Day 0. The monolayer of cells was cultured in Differential Basal Media + Differential Supplement A + 1% Penicillin-Streptomycin for two days. On days three and four, the monolayer was cultured with Differential Basal Media + Differential Supplement B + 1% Penicillin-Streptomycin. On days four through eight, the monolayer was cultured in Differential Basal Media + Differential Supplement C + 1% Penicillin-Streptomycin. On day eight, the monolayer was cultured in STEMDiff^TM^ Cardiomyocyte Maintenance medium + 1% Penicillin-Streptomycin and was maintained in this medium for at least twenty days (replenished every two days) to allow CMs to mature, as described.^4^

**Dissociation and Plating of Mature iPSC-CMs**

Spontaneously beating CMs were dissociated using STEMDiff^TM^ Cardiomyocyte Dissociation Kit (Stemcell Technologies, Vancouver, Canada; #05025) (document #DX21497). Briefly, iPSC-CMs were washed with D-PBS without CaCl_2_ and MgCl_2_ (Life Technologies, Carlsbad, CA) and incubated with STEMDiff^TM^ Cardiomyocyte dissociation media for 15 minutes at 37^o^C. Dissociated cardiomyocytes were re-plated at a lower density on coverslips coated with hESC-qualified Matrigel® (Corning, Bedford, MA) and resuspended in STEMdiff^TM^ Cardiomyocyte Support Medium at 37^o^C for 24 hours to allow the cells to attach. After 24 hours incubation, cells were maintained in STEMDiff^TM^ Cardiomyocyte Maintenance medium + 1% Penicillin-Streptomycin for at least 48 hours before being used for experiments.

**Ca^2+^** **Imaging of iPSC-CMs and Drug Treatment using EL20**

Cardiomyocytes (45±10 days old) were incubated for 30 minutes at 37°C in cell culture medium containing 10μM of the cytosolic Ca^2+^ dye Fluo-4-AM (Life Technologies, Carlsbad, CA). Following incubation, the indicator-containing medium was removed. Cells were washed once and incubated in cell culture medium for an additional 30 minutes at 37°C to allow for de-esterification of the indicator, followed by incubation in 1.8mM Ca^2+^ isotonic Tyrode (pH 7.4) for imaging. Ca^2+^ sparks were recorded using line-scan mode on a LSM880 confocal microscope (Carl Zeiss, Thornwood, NY). CaSpF was assessed with ImageJ software using SparkMaster plugin.^5^ Regions for analysis of CaSpF were selected from the portions outside the induced transients. SR Ca^2+^ load was measured as peak fluorescence to basal fluorescence after pacing followed by acute caffeine application (10mM) to release SR Ca^2+^. For drug assay studies, iPSC-CMs were incubated with drug for 30 minutes at 37^o^C following removal of excess indicator. EL20 was reconstituted in DMSO to a working stock solution of 0.1M, followed by a serial dilution for concentrations ranging from 0.5nM – 5μM. The half maximal inhibitory concentration (IC_50_) was calculated based on CaSpF measured in the presence of EL20 at different concentrations. All experiments were conducted at room temperature.

**Quantitative real-time PCR**

Total RNA was extracted from iPSCs and iPSC-CMs (45 ± 10 days old) using RNAqueous Micro Kit (Invitrogen, Carlsbad, CA; #AM1931). cDNAs were prepared from 1 μg RNA samples with reverse transcribed by iScript™ (Bio-Rad, Hercules, CA; #1708891), according to the manufacturer’s instructions. Quantitative real-time (qRT)-PCR was conducted in triplicate with 96-well PCR plates using iTaq SYBR Green I Supermix (Bio-Rad, Hercules, CA; #172-5120). For each sample, mRNA levels were normalized to TUBB1 levels, calculated using the cycle number method. Primers are listed in **Supplemental Table 1**. Results are shown in **Supplemental Table 2**.

**Immunofluorescence Staining**

Validation of CMs were done using immunostaining. Briefly, adherent cells were rinsed with cold 1× PBS and fixed with 4% paraformaldehyde solution for 10-15 minutes at room temperature (RT), permeabilized with 0.1% Triton X for 5 minutes, rinsed again with 1× PBS, and blocked overnight at 4°C with 10% normal goat serum (Thermo Fisher Scientific, Waltham, MA; #50062Z). Cells were incubated for 2 hours at RT with primary antibodies, then rinsed three times with 1× PBS to remove excess antibody. Cells were incubated with fluorescently conjugated secondary antibodies for 1 hour at RT in the dark. Cells were washed three more times with 1× PBS. Nuclear counterstaining was performed using Vectashield mounting media with DAPI (Vector Laboratories, Burlingame, CA; #H-1200). Immunofluorescent images of the stained cells were captured using a LSM880 confocal microscope (Carl Zeiss, Thornwood, NY). IF primaries and secondary antibodies are listed in **Supplementary Table 3**.

**Supplemental References**

**1.** Verhoeckx K, Cotter P, Lopez-Exposito I, et al., eds. *The Impact of Food Bioactives on Health: in vitro and ex vivo models*. Cham (CH)2015.

**2.** Mallone R, Mannering SI, Brooks-Worrell BM, et al. Isolation and preservation of peripheral blood mononuclear cells for analysis of islet antigen-reactive T cell responses: position statement of the T-Cell Workshop Committee of the Immunology of Diabetes Society. *Clin Exp Immunol.* 2011;163:33-49.

**3.** Brookhouser N, Zhang P, Caselli R, Kim JJ, Brafman DA. Generation and characterization of two human induced pluripotent stem cell (hiPSC) lines homozygous for the Apolipoprotein e4 (APOE4) risk variant-Alzheimer's disease (ASUi005-A) and healthy non-demented control (ASUi006-A). *Stem Cell Res.* 2018;32:145-149.

**4.** Hwang HS, Kryshtal DO, Feaster TK, et al. Comparable calcium handling of human iPSC-derived cardiomyocytes generated by multiple laboratories. *J Mol Cell Cardiol.* 2015;85:79-88.

**5.** Picht E, Zima AV, Blatter LA, Bers DM. SparkMaster: automated calcium spark analysis with ImageJ. *Am J Physiol Cell Physiol.* 2007;293:C1073-1081.

**Supplemental Table 1.** Forward and reverse primer sequences used for qPCR and validation of Sanger sequencing.

| **Gene** | **Forward** | **Reverse** |
| --- | --- | --- |
| *RYR2* | TAGATTTATAAGGGGCCTTG | GATTCTTCAGGGCTCGTAGT |
| *CAV1.2* | TGACATCGAGGGAGAAAACT | ACATTAGACTTGACTGCGGC |
| *SERCA* | GAGAACGCGCACACCAAGA | TTGGAGCCCCATCTCTCCTT |
| *PLN* | CTGCCAAGGCTACCTAAAAG | AGCTGAGCGAGTGAGGTATT |
| *NKX2.5* | ACCTCAACAGCTCCCTGACTCT | ATAATCGCCGCCACAAACTCTCC |
| *MYH7* | TTGAGTAGCCCAGGCACAG | TGAGGTCAAAAGGCCTGGTC |
| *TNNT2* | GACAGAGCGGAAAAGTGGGA | TCCTTGGCCTTCTCCCTCA |
| *TNNI3* | CCCTCACTGACCCTCCAAAC | GAGGTTCCCTAGCCGCATC |
| *NPPA* | AGTGGATTGCTCCTTGACGA | CCAAATGGTCCAGCAAATTCTTG |
| *SOX2* | TGAAGGAGCACCCGGATTAT | GTTCATGTGCGCGTAACTGT |
| *NANOG* | CAATGGTGTGACGCAGAAGG | CTGGATGTTCTGGGTCTGGT |
| *TUBB1* | CCGGACAGTGTGGCAACCAGATC | TGGCCAAAAGGACCTGAGCGAAC |
| WT or R176Q mutation | GTCACGTCACAAAACCCCAAG | AAGCCTGCCTGAACAACCTG |

**Supplemental Table 2.** qPCR results normalized to mRNA/TUBB1.

| **Marker** | **Control iPSC** | **Control iPSC-CM** | **P-Value** | **n** |  | **Marker** | **R176Q iPSC** | **R176Q iPSC-CM** | **P-Value** | **n** |
| --- | --- | --- | --- | --- | --- | --- | --- | --- | --- | --- |
| **Pluripotency markers** | | | | | | | | | | |
| **NANOG** | 0.10±0.01 | 0.002±0.001 | P<0.001 | 8 |  | **NANOG** | 0.10±0.02 | 0.002±0.002 | P<0.001 | 7 |
| **SOX2** | 0.060±0.008 | 0.006±0.002 | P<0.001 | 8 |  | **SOX2** | 0.40±0.08 | 0.010±0.003 | P<0.001 | 8 |
| **Cardiomyocyte markers** | | | | | | | | | | |
| **NPPA** | 1e^-6^±1e^-5^ | 0.110±0.002 | P=0.018 | 8 |  | **NPPA** | 1e^-8^±1e^-5^ | 0.018±0.008 | P<0.001 | 6 |
| **TNNI3** | 1e^-6^±1e^-5^ | 0.012±0.004 | P=0.048 | 8 |  | **TNNI3** | 1e^-6^±8e^-6^ | 0.002±0.001 | P=0.012 | 6 |
| **TNNT2** | 0.002±0.001 | 0.418±0.090 | P=0.049 | 8 |  | **TNNT2** | 0.017±0.001 | 0.287±0.014 | P=0.0014 | 8 |
| **NKX2.5** | 1e^-6^±2e-^5^ | 0.025±0.008 | P=0.049 | 6 |  | **NKX2.5** | 1e^-6^± 2e-^6^ | 0.028±0.014 | P=0.027 | 6 |
| **Ca^2+^ Handling markers** | | | | | | | | | | |
| **PLN** | 0.070±0.007 | 0.215±0.042 | P=0.020 | 8 |  | **PLN** | 0.0008±0.0004 | 0.116±0.047 | P<0.001 | 6 |
| **LTCC** | 1e^-6^±6e^-5^ | 0.005±0.001 | P=0.027 | 7 |  | **LTCC** | 0.0009±0.0002 | 0.005±0.001 | P<0.001 | 6 |
| **RyR2** | 0.004±0.001 | 0.016±0.005 | P=0.049 | 7 |  | **RyR2** | 0.005±0.001 | 0.011±0.004 | P=0.026 | 6 |

**Supplemental Table 3.** Primary antibodies used for immunocytochemistry

| **Primary Antibody Name** | **Source** | **Dilution** | **Company (#Catalogue)** | **Lot #** |
| --- | --- | --- | --- | --- |
| N-terminal actin | Polyclonal rabbit | (1:100) | Sigma-Aldrich Co., St. Louis, MO 63102, USA (#A2103) | n/a |
| Sarcomeric α-actinin | Monoclonal mouse | (1:100) | Sigma-Aldrich Co., St. Louis, MO 63102, USA (#A7811) | 097K4758 |
| Voltage-dependent L-type Ca^2+^ channel subunit α1C (CaV1.2) | Polyclonal rabbit | (1:100) | Alomone Labs., Jerusalem BioPark (JBP), Hadassah Ein Kerem, P.O.Box 4287 Jerusalem 9104201, Israel (#ACC-003) | ACC003an6002 |
|  |  |  |  |  |
| Cardiac troponin T | Monoclonal mouse | (1:100) | Thermo Fisher Scientific., Waltham, MA 02451, USA (#MA5-12960) | TC2540252C |
| Atrial isoform of the myosin light chain 2 (MLC2a) | Monoclonal mouse | (1:100) | Synaptic Systems GmHbH., 37079 Goettingen, Germany (#311 011) | 311011/1-35 |
| Myomesin, | Monoclonal mouse | (1:100) | EMD Millipore Corporation., Temecula, CA 92590, USA (#MABT24) | 2975261 (clone 5D11) |
| Myosin light chain 2 (MYL2) | Polyclonal rabbit | (1:100) | Proteintech Group, Inc., Rosemont, IL 60018, USA (#10906-1-AP) | 2975261 |
| NK2 homeobox protein 5 (Nkx-2.5) | Monoclonal mouse | (1:100) | Santa Cruz Biotechnology Inc., Dallas, Texas 75220, USA (#sc-376565) | C0518 (clone A3) |
| Phospholamban | Monoclonal mouse | (1:100) | Thermo Fisher Scientific., Waltham, MA 02451, USA (# MA3-922) | PJ201791 (clone 2D12) |
| Ryanodine receptor | Mouse monoclonal | (1:100) | Thermo Fisher Scientific., Waltham, MA 02451, USA (# MA3-916) | SK253776 (clone C3-33) |

**
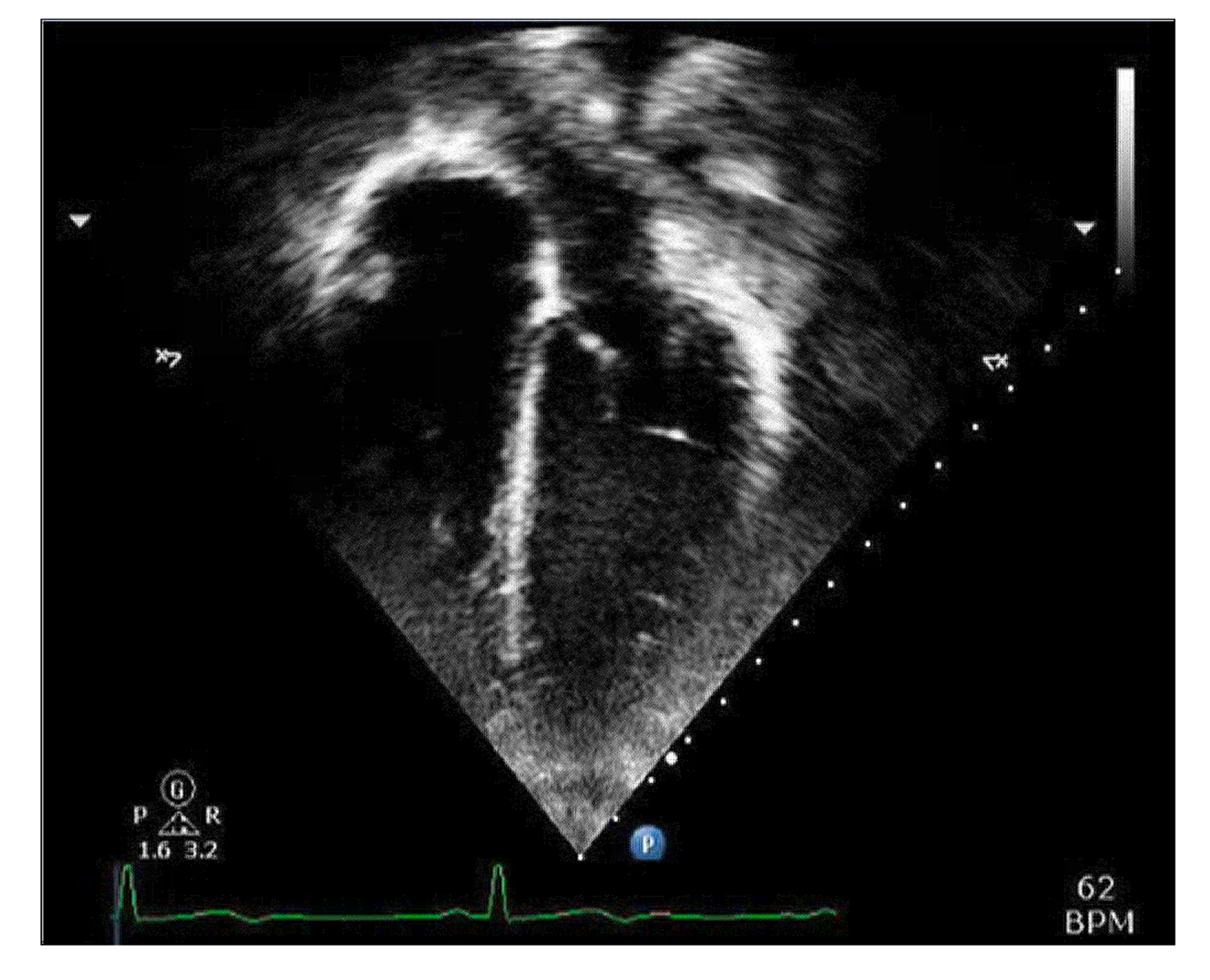
**

**Supplemental Figure 1.** Echocardiographic image of the proband’s heart. Echocardiogram image of 14-year-old proband who was found to have a structurally normal heart with normal biventricular function.


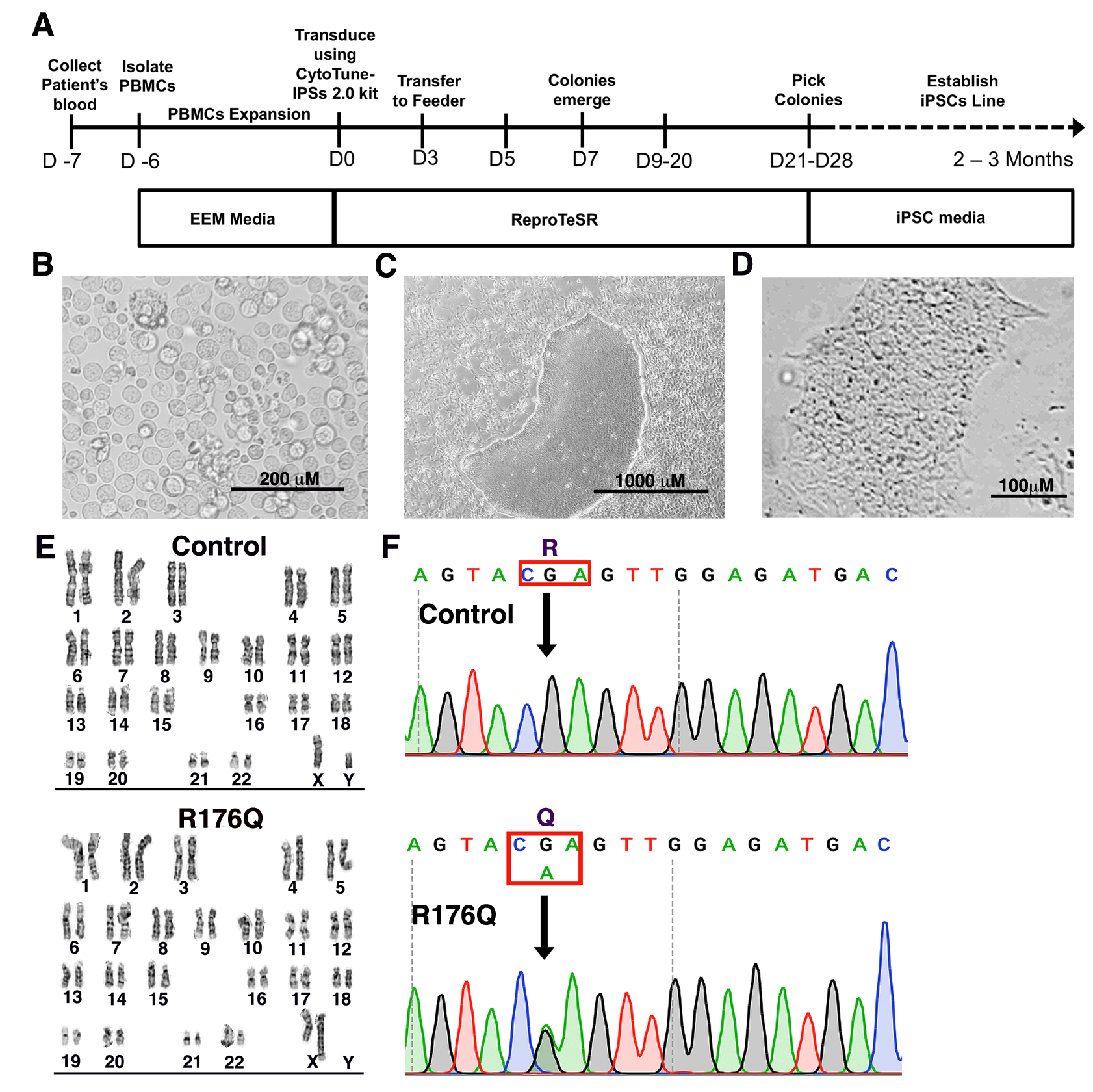


**Supplemental Figure 2.** Generation and characterization of patient-derived iPSCs. **(A)** Timeline showing the steps involved with establishing the iPSC lines from isolated PBMCs**. (B)** Bright field (BF) image showing isolated PBCMs growing in culture. Scale bar, 200 μM. **(C)** BF image of iPSC colony emerging nine days after transduction. Scale bar, 1000 μM. **(D)** BF image showing typical morphology of an iPSC colony. Scale bar, 100 μM. **(E)** Normal karyotype analysis of iPSCs derived from (top) mutation negative relative (control) and (bottom) R176Q proband. **(F)** Sanger sequencing conformation of R176Q mutation in the proband vs. control, highlighting G to A substitution by Sanger sequencing analysis.

**
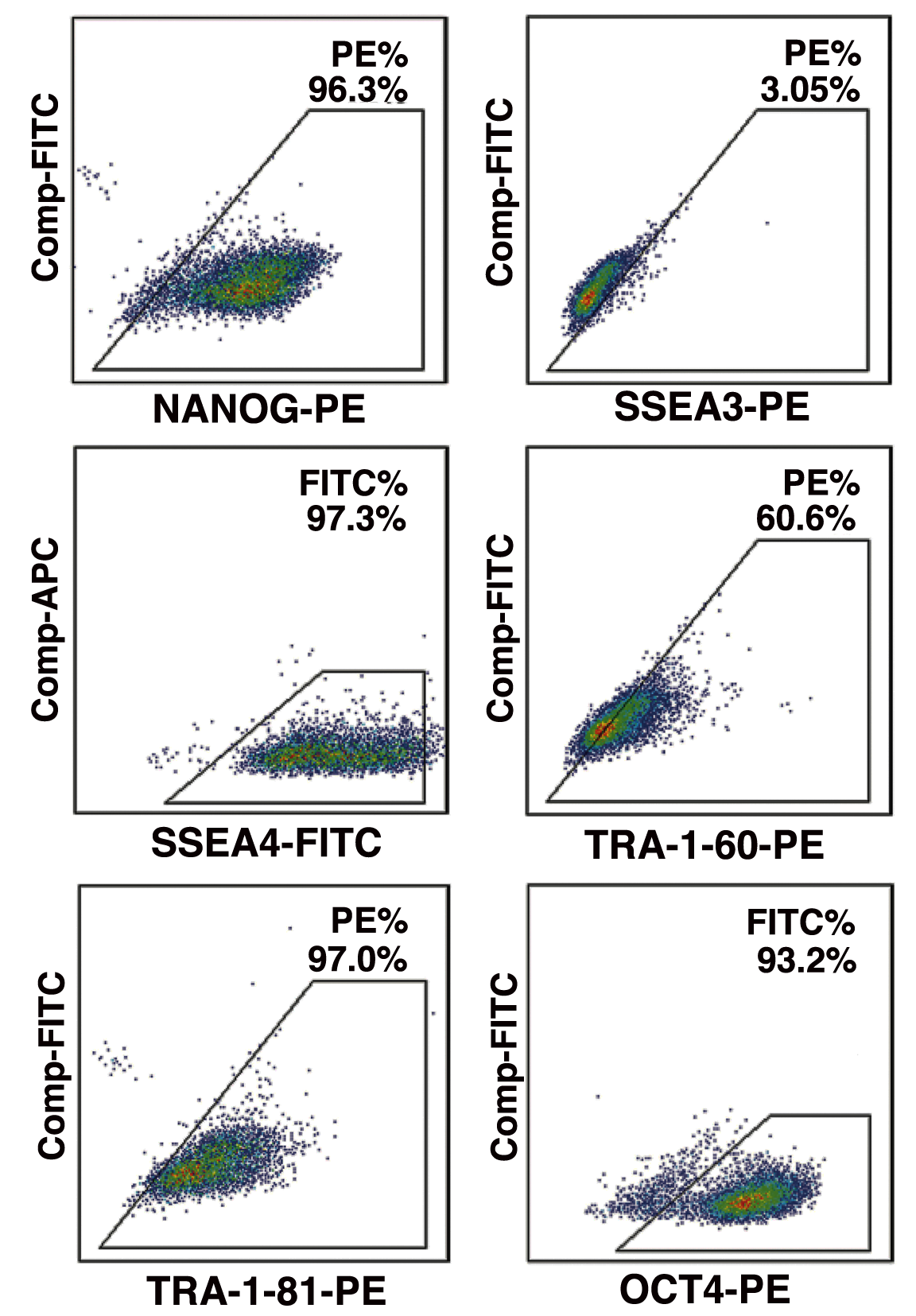
**

**Supplemental Figure 3.** FACS analysis of R176Q iPSC line. Representative 2D diagrams of flow cytometry plotting analysis of CD44 (FITC) and CD133 (PE) expression in R176Q iPSC line showing pluripotency of the iPSC lines. Dead cells, cell debris and doublets were gated out. Compensation for background fluorescence was performed by measuring target signals of single color controls and negative controls.

**
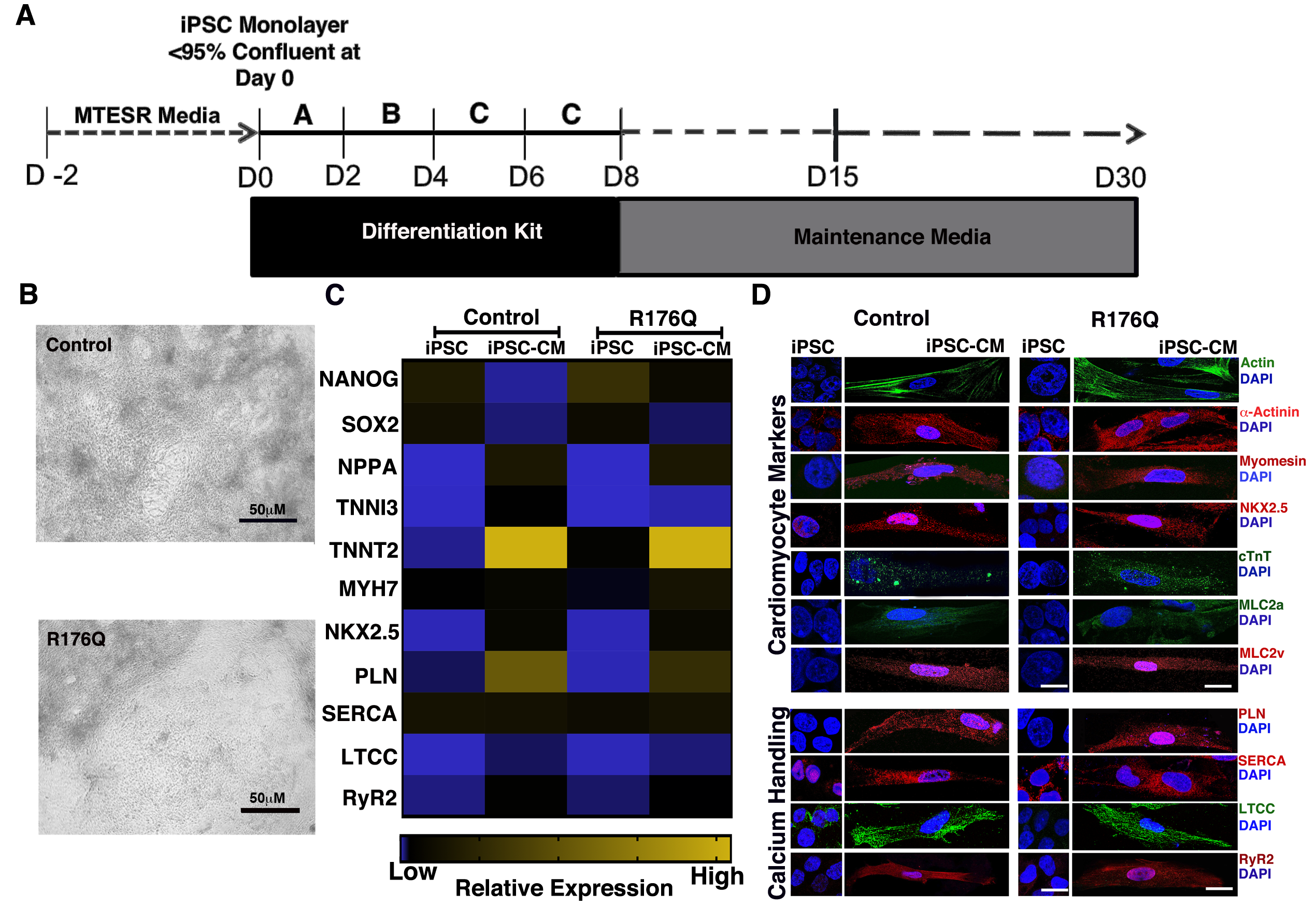
**

**Supplemental Figure 4.** Generation and characterization of iPSC-derived CMs. **(A)** Timeline of CM using STEMdiff^TM^ CM differentiation kit. **(B)** Phase contrast images of (i) control and (ii) R176Q iPSC-CMs in confluent monolayers. **(C)** Heat map showing successfully differentiated CMs with increased levels of Ca^2+^-handling markers and decreased levels of stem cell markers. **(D)** Immunostaining of iPSC-CMs shows positive staining of cardiomyocyte markers that do not express in iPSCs.


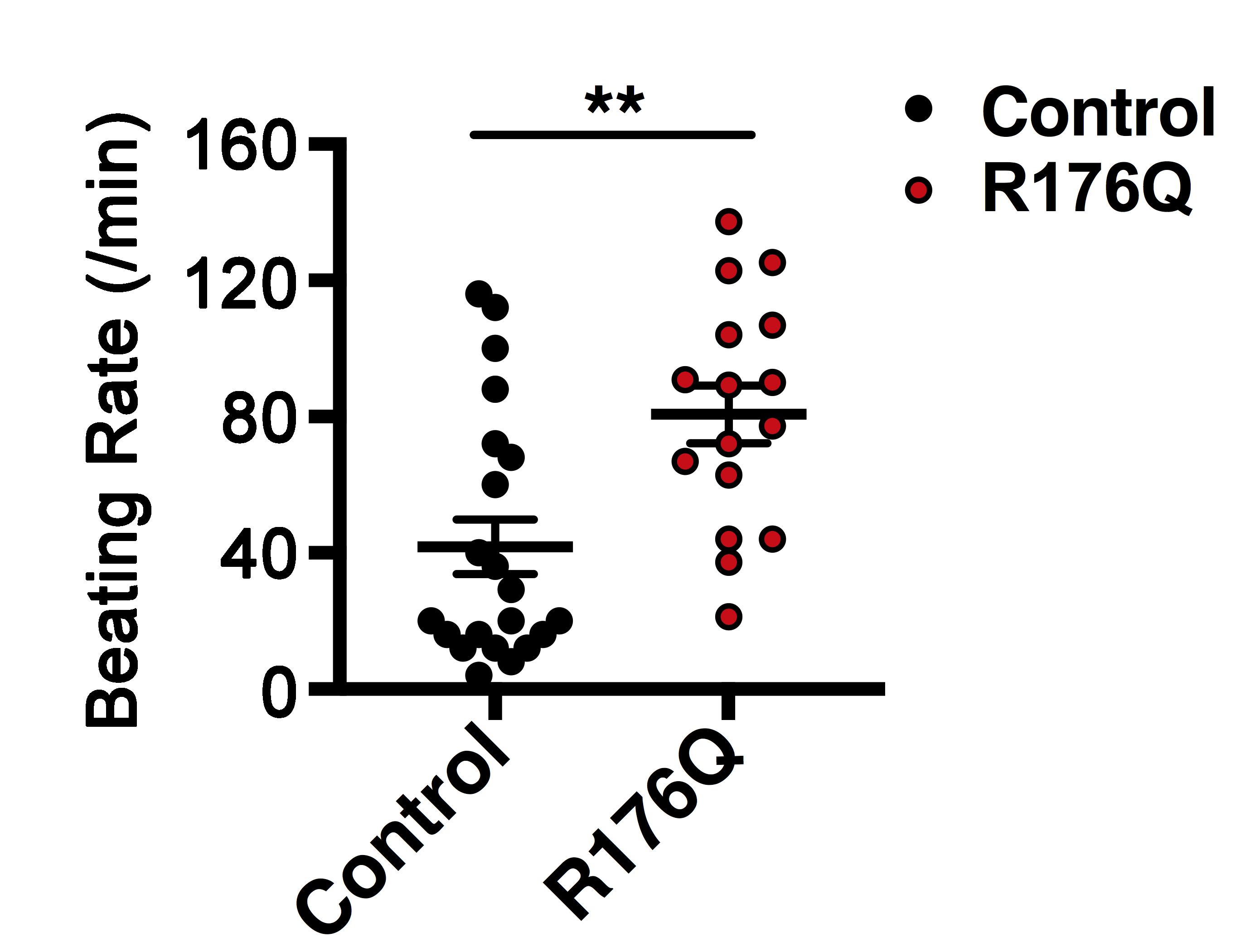


**Supplemental Figure 5.** Spontaneous beating rate of control and R176Q iPSC-CMs. **P<0.01.


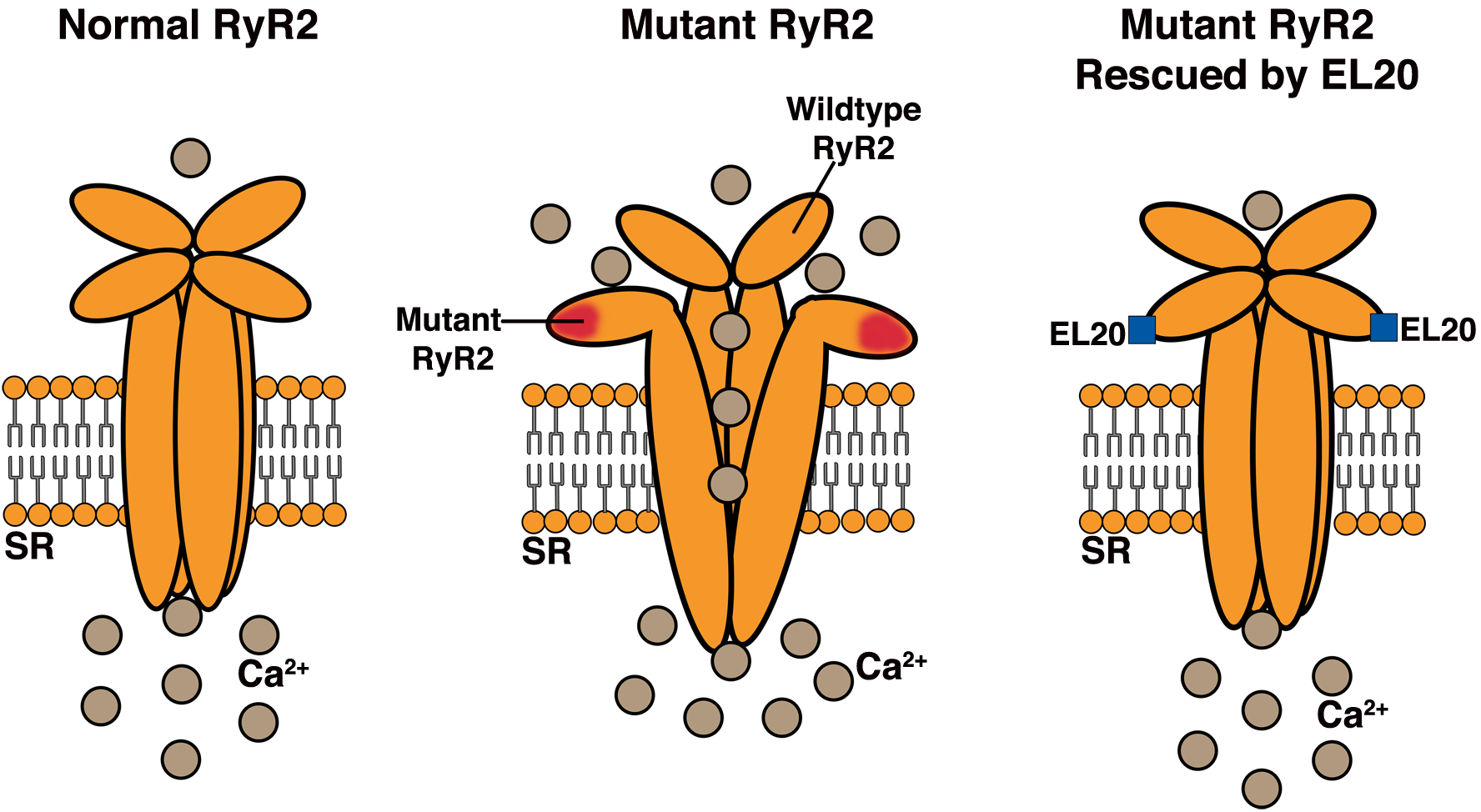


**Supplemental Figure 6.** EL20 effect on normal (wildtype) and CPVT mutant RyR2 channels. Schematic representation of (*left*) normal RyR2 channels, (*middle*) mutant RyR2 channel prone to leakage of Ca^2+^ from the sarcoplasmic reticulum, and (*right*) normalization of mutant RyR2 channels as a result of EL20 inhibitory effects on channel activity.
